# Development of a New Instrument for Depression With Cognitive Diagnosis Models

**DOI:** 10.3389/fpsyg.2019.01306

**Published:** 2019-06-04

**Authors:** Daxun Wang, Xuliang Gao, Yan Cai, Dongbo Tu

**Affiliations:** School of Psychology, Jiangxi Normal University, Nanchang, China

**Keywords:** psychological measurement, cognitive diagnosis models, symptom criteria-level information, psychometrics, questionnaires, depression

## Abstract

Most existing instruments for depression are developed based on classical test theory, factor analysis, or sometimes, item response theory, and focus on the accurate measurement of the severity of depressive disorder. Nevertheless, they tend to be less useful in supporting the decision based on *ICD-10* or *DSM-5* because of the lack of detailed information for symptoms. To gain rich and valid information at the symptom level, this article developed a depression test under the framework of cognitive diagnosis models (CDMs), referred to as CDMs-D. A total of 1,181 individuals were finally recruited and their responses were used to examine the psychometric properties of CDMs-D. After excluding poor items for statistical reasons (e.g., low discrimination, poor model-fit or having DIF), 56 items were included in the CDMs-D. The CDMs-D measures all ten symptom criteria for depression defined in *ICD-10* and covers five domains of depression defined by Gibbons et al. (2012). Comparing with the existing self-report measures (such as PHQ-9, SDS, CES-D and so on), a distinguishing feature of the CDMs-D is that it can provide both overall information about the severity of depressive disorder and the assessment information about specific symptoms, which could be useful for diagnostic and interventional purposes.

## Introduction

Depression is one of the most common and prevalent psychological and behavioral disorders. By the year 2020, depression accounting for 5.7% of the total burden of the disease (Dennis et al., 2016) will be the second disease leading to disability and death with the exception of coronary heart disease according to the World Health Organization (Dennis and Hodnett, 2014). A number of self-report inventories have been developed to assess the severity of the depressive disorder, such as the Self-Rating Depression Scale (SDS; Zung, 1965), the Center for Epidemiologic Studies Depression Scale (CES-D; Radloff, 1977) and the Beck Depression Inventory (BDI; Beck et al., 1961).

Despite having sound psychometric properties and being widely used, they are also some rooms for improvement. For example, most existing self-report inventories are unidimensional and yield overall scores indicating the severity of the depressive disorder on a continuum. To determine whether it is a mild, moderate or severe depression, the scores are compared with some cutoffs. This procedure is straightforward, but it is not informative given that they cannot provide all symptom-level information of depression defined in the 10th revision of the *International Classification of Diseases* (*ICD-10;* World Health Organization [WHO], 2010) or the 5th edition of the *Diagnostic and Statistical Manual of Mental Disorders* (*DSM-5*; American Psychiatric Association [APA], 2013). However, these symptom-level information of depression are helpful for assessment, screening, monitoring and even intervention of depression. For example, as shown in Table 1, the *ICD-10* groups the symptoms of depression into two sets: typical symptoms and common symptoms and its diagnostic thresholds are specified in terms of the number of symptoms required from each of the two sets. More specially, for the mild depressive episode, two typical symptoms and two common symptoms are required; for the moderate depressive episode, two typical symptoms and at least three common symptoms are required; for the severe depressive episode, all three typical symptoms are present and at least four common symptoms of severe intensity are required. As known, this type assess for depression is more informative than the score cutoffs of conventional inventories given that the patients with the same score may have very different symptoms which can provide more information for screening or treatment.

**Table 1:** Symptom criteria for depression defined in the *DSM-5* and *ICD-10.*

*DSM-5*	*ICD-10*
(1) Depressed mood (2) Markedly diminished interest or pleasure (3) Significant weight loss (4) Insomnia or hypersomnia (5) Psychomotor agitation or retardation (6) Fatigue or loss of energy (7) Feelings of worthlessness or excessive or inappropriate guilt (8) Diminished ability to think or concentrate, or indecisiveness (9) Recurrent thoughts of death (not just fear of dying), recurrent suicidal ideation without a specific plan or a suicide attempt or a specific plan for committing suicide.	**Typical symptom criteria** (1) Depressed mood (2) Loss of interest and enjoyment (3) Increased fatigability **Common symptom criteria** (4) Reduced concentration and attention (5) Reduced self-esteem and self-confidence (6) Ideas of guilt and unworthiness (even in a mild type of episode) (7) Bleak and pessimistic views of the future (8) Ideas or acts of self-harm or suicide (9) Disturbed sleep (10) Diminished appetite

Form a very different perspective, this study aims to develop a new measure of depression that is aligned with the *ICD-10* to provide more information for the screening and monitoring of depression under the framework of cognitive diagnosis models (CDMs; see Rupp et al., 2010). Compared with the factor analysis technique or item response theory (IRT), the CDMs provide an alternative psychometric framework for test development, psychometric analyses, and score reporting. Although most of research on CDMs lies in the field of education measurement, researchers have been recently aware of their usefulness in psychological disorder assess for identifying individuals’ disorder or symptom profiles (e.g., Jaeger et al., 2006; Templin and Henson, 2006; de la Torre et al., 2017). Specifically, it is possible to infer about whether each of the symptom criteria has been satisfied or not from patients’ responses to items in an instrument. This information can be useful for screening (or intervening) depressive disorder or other psychological disorders based on the *ICD-10* or *DSM-5*. In addition, compared with factor analysis, CDMs allow latent variables (i.e., symptom criteria) to interact when producing manifest item responses and thus are more flexible.

In specially, the goal of this study is twofold. First, this study develops a depression test under the framework of CDMs (CDMs-D) based on the *ICD-10* under the CDMs framework, which may be used to assess, screen and monitor depression. Different from the existing self-report questionnaires for depression, the CDMs-D can assess how likely each of the symptom criteria of depression in the *ICD-10* has been met for each patient, and estimate the probability of having mild, moderate and severe depressive episode using the *ICD-10* diagnostic criteria. Second, this study aims to provide an illustration about how CDMs can be used to develop instruments, assess psychometric properties using the *ICD-10* system. This could serve as an example for researchers willing to develop instruments for other psychological disorders using CDMs to provide patient outcomes consistent with *ICD-10* or *DSM-5* criteria.

## Materials and Methods

### Diagnosis System of Depression

Currently, two famous diagnosis systems of depression are *ICD-10* and *DSM-5*, which are both commonly acceptable and used to guide the diagnosis of depression in clinical practice. There are eight common symptom criteria of depressive disorder in *ICD-10* and *DSM-5* (see Table 1). In this article, the symptom criteria for depression in the *ICD-10* were used in that the *ICD-10* distinguishes three types of depression (mild, moderate or severe/major depression) and thus could provide more information.

### Cognitive Diagnosis Models

In the context of CDMs, 10 symptom criteria of depression in *ICD-10* are treated as latent variables that need to be measured, each with two outcomes – 1 and 0, representing presence and absence, respectively. Based on individuals’ responses to items of the CDMs-D and the aforementioned item and symptom association matrix, CDMs estimate the symptom profile for each individual. For example, if the symptom profile for an individual is estimated to be (0,1,1,0,0,0,1,1,0,0), this individual is said to meet symptom criteria 2, 3, 7, and 8. In addition, CDMs can also estimate the probability of an individual meets each criterion.

An array of CDMs can be found in the literature (Rupp et al., 2010). In this study we adopt the generalized deterministic input, noisy, “and” gate (G-DINA; de la Torre, 2011) model framework because (1) it is one of the most general CDMs with many applications and (2) it is very flexible and subsumes many reduced CDMs. The G-DINA model, like most other CDMs, is a psychometric model specifying how individuals respond to each item given their symptom criteria. Take item “I feel worthless and ashamed” as an example, which measures (C5) “reduced self-esteem and self-confidence” and (C6) “ideas of guilt and unworthiness.”

Let α = (α_1_,α_2_) denote the profile of these two criteria. Based on the G-DINA model (de la Torre, 2011), the probability of endorsement on this item given the symptom profile α can be written by P(α) = ϕ_0_ + ϕ_1_α_1_ + ϕ_2_α_2_ + ϕ_12_α_1_α_2_. More specifically, for α = (0,0), where both symptoms are absent, the corresponding endorsement probability isP(0,0) = ϕ_0_; for α = (1,0), where symptom C5 is present but C6 is absent, the corresponding endorsement probability is P(1,0) = ϕ_0_ + ϕ_1_, where ϕ_1_ is the effect of symptom C5; for α = (0,1), where symptom C5 is absent but C6 is present, the corresponding endorsement probability is P(0,1) = ϕ_0_ + ϕ_2_, where ϕ_2_ is the effect of symptom C6; and for α = (1,1), where both symptoms are present, the corresponding endorsement probability is P(1,1) = ϕ_0_ + ϕ_1_ + ϕ_2_ + ϕ_12_, where ϕ_12_ is the interaction effect of symptoms C5 and C6.

Although the G-DINA model considers all possible interactions among measured symptom criteria, researchers may have some assumptions about how symptom criteria produce item responses. For example, the deterministic inputs, noisy “and” gate (DINA) model assumes that the endorsement probability will not increase unless all measured symptom criteria have been present. This model can be obtained, for the aforementioned example, by setting ϕ_1_ = ϕ_2_ = 0 such that P(0,0) = P(1,0) = P(0,1) = ϕ_0_ and P(1,1) = ϕ_0_ + ϕ_12_. In contrast, the deterministic inputs, noisy “or” gate (DINO; Templin and Henson, 2006) model assumes that a high endorsement probability is expected if any of the measured symptom criteria is present. This model can be obtained by setting ϕ_1_ = ϕ_2_ = −ϕ_12_ such that P(0,0) = ϕ_0_ and P(1,0) = P(0,1) = P(1,1) = ϕ_0_ + ϕ_1_. In addition, the addictive CDM (A-CDM; de la Torre, 2011), linear logistic model (LLM; Maris, 1999) and reduced reparameterized unified model (rRUM; Hartz et al., 2002) can be obtained by assuming all symptom criteria contribute independently and uniquely without interaction effects. For more details on these models, please refer to de la Torre (2011).

### Development of Cognitive Diagnostic Test for Depression (CDMs-D)

The CDMs-D is designed to be a self-report instrument and the ultimate goal is to infer whether an individual has satisfied each of the symptom criteria of depression defined in the *ICD-10* and the probability of having mild, moderate and severe depressive episode from his or her responses. The CDMs-D primitively included 89 items which were carefully chosen according to the depression symptom criteria in the *ICD-10* from several self-rating inventories, including the Zung’s SDS, the CES-D (Radloff, 1977), the Patient Health Questionnaire (PHQ-9; Kroenke et al., 2001), the Hospital Anxiety Depression Scale (HADS), Carroll’s Depression Scale (CDS; Carroll et al., 1981), Minnesota Multiphasic Personality Inventory (MMPI; Hathaway and McKinley, 1942), the Brief Depression Scale (BDS; Koenig et al., 1992), the Geriatric Depression Scale (GDS), the Edinburgh postnatal depression Scale (EPDS; Cox et al., 1987) and the Adolescents Depression Emotion Self-assessment Scale (ADESC; Huang et al., 2004). The chosen 89 items measure all ten depression symptom criteria in *ICD-10* and involve five domains of depression defined by Gibbons et al. (2012), namely, mood (14 items), cognition (30 items), behavior (21 items), somatic complaints (17 items) and ideas or acts of suicidality (7 items). Items were revised to refer to the previous 2-week period and to have consistent response categories. Each item measures at least one depression symptom criterion in *ICD-10*.

The way of an individual responding to an item can be reasonably assumed to be influenced by whether she/he has satisfied some symptom criteria. For example, an individual may agree with that “I feel worthless and ashamed” if she/he has “reduced self-esteem and self-confidence” (C5) or “ideas of guilt and unworthiness” (C6) and agree with that “I wish to be dead” if she has “ideas or acts of self-harm or suicide” (C8). To make inference as to whether individuals have satisfied each symptom criterion from their item responses, an item by symptom association matrix giving which symptom criteria may influence individuals’ item responses needs to be developed in advance. For CDMs-D, the item and symptom association matrix was constructed using the Delphi method with three experts (two psychotherapists with more than 5 years of clinical experience and one with 5-year research experience in the measurement of depression). Table 2 gives some exemplary items and their association with symptom criteria, where entry 1 indicates a symptom criterion is measured by the item and entry 0 indicates not. On average, each item measures 1.67 symptom criteria, and each criterion is measured by 14.9 items.

**Table 2:** Exemplary items in CDMs-D.

Items (abbreviated content)	Domain	Q-matrix									
		C1	C2	C3	C4	C5	C6	C7	C8	C9	C10
Worthlessness and shame	Cognition	0	0	0	0	1	1	0	0	0	0
Feeling unhappy	Mood	1	0	0	0	0	0	0	0	0	0
Everything is laborious	Behavior	0	0	1	0	0	0	0	0	0	0
Wish to be dead	Suicidality	0	0	0	0	0	0	0	1	0	0

*Criteria C1, C2, and C3 represent three typical symptoms; criteria C4–C10 represent seven common criteria in ICD-10.*

### Participant Sample

Participants include healthy individuals and patients with depression. Depressive patients, who were being treated for depression, were recruited from eight health centers and hospitals in seven provinces/cities of China, whereas the healthy individuals were mainly from colleges and social groups. The selected seven provinces/cities distribute in east, south, west, and north area of China and covers mainly area of China. The final selection of both depressive patients and healthy individuals were recruited according to the following exclusion criteria: history of psychosis, schizoaffective disorder, or schizophrenia; organic neuropsychiatric syndrome, such as dementia and Parkinson disease; drug or alcohol dependence over the past 3 months, but not excluded patients with episodic abuse related to mood episodes. The study also had exclusion criteria to screen the healthy individuals: history of psychosis, schizoaffective disorder, or schizophrenia; any diagnosis or treatment for psychiatric illness over the past year. The study was approved by the medical ethics committees of participating health center and hospitals, and all participants were provided written informed consent.

A total of 1,286 samples were recruited, among which 92 samples had large missing data in the questionnaire and 13 samples met the exclusion criteria. After excluding the above 105 samples, the final selected participant sample was consisted of 1,181 individuals aged from 18 to 80 with mean = 31.8 (*SD* = 12.92) based on the above exclusion criteria for this study. The number of depressive patients and healthy individuals were 488 (41.3%) aged from 18 to 80 with mean = 36.8 (*SD* = 14.9), and 693 (58.7%) aged from 18 to 57 with mean = 28.36 (*SD* = 10.03), respectively.

The total sample was randomly split into two subsamples. One of the resulting two subsamples was half of the overall sample and used as a calibration sample (*N_1_* = 591) to develop the CDMs-D. The other half sample was used as the cross validation sample (*N_2_* = 590) to verify the CDMs-D and investigate the reliability and validity of CDMs-D. Detailed demographic information was documented in Table 3.

**Table 3:** Demographic characteristics of depressive disorder patients and healthy individuals.

Characteristic	Calibration Sample, % (*N*_1_ = 591) Total (Male/Female)	Validation Sample, % (*N*_2_ = 590) Total (Male/Female)
**Gender**		
Male	46.4	47.8
Female	53.6	52.2
Age, years old		
18–29	62.1 (46.3/53.7)	58.5 (49.9/50.1)
30–39	13.5 (55/45)	12.2 (38.9/61.1)
40–49	12.5 (46.6/53.4)	12.7 (50.7/49.3)
50–59	8.8 (42.3/57.7)	12.0 (45.1/54.9)
≥60	3.0 (27.8/72.2)	4.6 (34.6/65.4)
**Education**		
Some high school or < 9th grade	20.6 (43/57)	20.5 (38.3/61.7)
High School diploma or GED	17.3 (38.9/61.1)	19.5 (47/53)
College graduate	52.5 (50.6/49.4)	51.2 (50.2/49.8)
Graduate or professional degree	9.6 (42.1/57.9)	7.1 (52.4/47.6)
**Area**		
Rural	48.4 (52.3/47.7)	48.1 (53.9/46.1)
Urban	51.4 (46.2/53.8)	50.2 (41.4/58.6)
other	0.2 (0/1)	1.7 (45.4/54.6)
**Group**		
Healthy	58.2 (47.4/52.6)	59.2 (50.9/49.4)
Depression	41.8 (45.5/54.5)	40.8 (42.7/57.3)

### Statistical Analysis

The calibration sample (*N_1_* = 591) was used in this step to develop the CDMs-D.

#### Item Analysis

Selecting suitable CDM is deemed to be a critical procedure for making valid inferences. Although a number of CDMs are available, it’s not always clear which model should be chosen for a given data set. The Wald test (de la Torre, 2011; Ma et al., 2016) was proposed to evaluate whether the reduced CDM can be replaced by the saturated CDM without significant loss in model-fit (de la Torre, 2011), and the results of Ma et al. (2016) indicated that the chosen CDMs via the Wald test performed better than the saturated CDM in terms of estimation of person parameter. In this study five special or reduced CDMs were considered, which were the deterministic inputs, noisy “and” gate model (DINA; Junker and Sijtsma, 2001), the deterministic input, noisy “or” gate model (DINO; Templin and Henson, 2006), the addictive CDM (A-CDM; de la Torre, 2011), the linear logistic model (LLM; Maris, 1999) and the reduced reparameterized unified model (RRUM; Hartz et al., 2002). The Wald test was carried out for items measuring more than one criterion in that all CDMs are equivalent for single criterion items.

After choosing the suitable model for each item, the *S-X^2^* item fit statistic (Orlando and Thissen, 2000) was used to assess the adequacy of item fit, followed by the detection of the differential item functioning (DIF) for different groups (e.g., female and male, rural and urban) using the Wald statistic (Hou et al., 2014). Then, the discrimination index (*Disc*) suggested by de la Torre (2008) was calculated to assess item quality. The above statistical analyses were conducted step by step.

In Step 1, the item fit analysis was carried out via *S-X^2^* item fit statistic and items with poor fit (*p*-value of *S-X^2^* less than 0.01) were deleted from the CDMs-D. In Step 2, for the remainder items in Step 1, DIF analysis was employed and items with DIF were excluded from the CDMs-D. In Step 3, for the remainder items in Step 2, we assessed item discrimination and items with low discrimination (*Disc* < 0.4) were deleted. That is to say, any item that had low discrimination (*Disc* < 0.4), had DIF or fitted to the data inadequately was removed from the CDMs-D. This procedure (three steps) was repeated until no item was deleted. The GDINA R package (Ma and de la Torre, 2016) and Custom-written code in R (R Core Team, 2016) were used for analyses.

Then the cross validation sample (*N_2_* = 590) was used to re-analyze and validate the remained items selected by the calibration sample (*N_1_* = 591). At this step the items that had low discrimination, DIF or poor item fit would be also deleted form the final CDT-T.

#### Reliability and Validity

The analysis of both the reliability and validity were carried out for the final CTD-D after above item analysis and item selection only with the cross validation sample (*N_2_* = 590). Under the framework of cognitive diagnosis, the symptom-level classification consistency and accuracy indices (Cui et al., 2012; Templin and Bradshaw, 2013) based on CDMs were investigated for CDMs-D. Criterion-related and convergent validity were then assessed by the coefficients of correlation between the CDMs-D and the SDS and individual’s self-reported depression and the. Content validity was examined as well in terms of whether the CDMs-D measures all the depression symptoms defined in *ICD-10* and covers all the domains of depression defined by Gibbons et al. (2012).

#### Depression Assessment

The posterior probability of satisfying symptom criterion *k* for individual *i* can be calculated as in

$$P(\alpha_{k}|{\text{X}}_{i})=\sum_{\forall w:\alpha_{\mathrm{wt}}=1}P(\alpha_{k}|{\text{X}}_{i}),$$

where P(α_*w*_|X_*i*_) is the posterior probability of having symptom profile α_*w*_ for individual*i*. Based on the posterior probability of satisfying each symptom criterion, we can calculate the probability of having each symptom criteria profile and the probability of being considered as mild, moderate or severe depression.

## Results

### Item Analysis of the CDMs-D

Using the aforementioned item analysis procedure, 31 items were deleted with the calibration sample (*N_1_* = 591). Specifically, 20 of them had low discrimination index (*Disc* < 0.4), 5 were DIF items and 10 showed poor item-fit (*p* < 0.01). After that, the remained 58 items were analyzed with the cross validation sample (*N_1_* = 590). Results showed that 56 items had high discrimination, good item-fit and no DIF except two items with low item fit. Therefore, the final CDMs-D had 56 items, which are given in Table 4. The CDMs-D measures all ten symptom criteria for depression defined in the *ICD-10* and involves five domains of depression which are mood (7 items), cognition (23 items), behavior (10 items), somatic complaints (9 items) and ideas or acts of suicidality (7 items). The number of items measuring each symptom criteria varies from 4 to 22 with an average of 10.4. In addition, there are 17, 31, 7, and 1 item (s) measuring 1, 2, 3, and 4 symptom criteria respectively with an average of 1.85 symptom criteria per item.

**Table 4:** Final items of the CDMs-D.

Item No.	Item abbreviation	Selected model	Discr.	Item-fit		DIF1		DIF2		Domain of depression
				*S-X^2^*	*p*	*Wald*	*p*	*Wald*	*p*	
Item 1	Lose daily life ability	RRUM	0.57	55.89	0.232	0.90	0.827	1.24	0.743	Behavior
Item 2	Talking slow and dully	DINA	0.54	62.32	0.096	3.07	0.216	0.39	0.822	Behavior
Item 7	Unable to do things	DINA	0.58	56.06	0.227	1.68	0.431	0.48	0.788	Behavior
Item 8	Broken down	ACDM	0.94	40.41	0.774	0.26	0.992	2.78	0.595	Behavior
Item 9	Full of energy	ACDM	0.60	42.02	0.750	3.08	0.380	4.32	0.229	Behavior
Item 10	Talk less	ACDM	0.58	38.38	0.863	2.26	0.520	1.13	0.770	Behavior
Item 11	Confidence of doing everything	ACDM	0.51	50.73	0.366	2.89	0.576	1.67	0.796	Cognition
Item 12	Loneliness feelings	GDINA	0.44	57.62	0.214	0.45	0.797	4.25	0.120	Cognition
Item 13	Clear mind	GDINA	0.62	37.42	0.906	0.75	0.687	4.04	0.133	Cognition
Item 14	Unchanged working/studying ability	ACDM	0.60	51.05	0.393	1.08	0.781	2.54	0.468	Cognition
Item 15	Deal with daily life easily	RRUM	0.54	65.04	0.062	1.53	0.675	5.11	0.164	Cognition
Item 16	Future hopeless feelings	ACDM	0.82	46.62	0.530	2.89	0.576	0.56	0.967	Cognition
Item 17	Unpopularity feelings	GDINA	0.56	40.00	0.843	1.27	0.530	0.43	0.806	Cognition
Item 18	Future hopeful feelings	RRUM	0.76	51.25	0.386	1.75	0.625	3.27	0.351	Cognition
Item 19	Loser feelings	ACDM	0.87	42.40	0.736	2.89	0.408	5.40	0.145	Cognition
Item 20	Failure in life	ACDM	0.84	39.60	0.829	0.70	0.873	1.53	0.676	Cognition
Item 21	Worthiness	ACDM	0.63	48.95	0.475	0.26	0.968	1.60	0.660	Cognition
Item 22	Worthlessness and shame	ACDM	0.89	40.20	0.810	0.96	0.811	0.01	1.000	Cognition
Item 23	Guilty feelings	GDINA	0.57	62.77	0.106	0.16	0.924	0.94	0.626	Cognition
Item 24	Reading disorder	ACDM	0.58	57.90	0.180	4.82	0.186	3.61	0.307	Cognition
Item 25	Feelings of being talking about	DINO	0.40	59.59	0.143	0.25	0.882	2.15	0.342	Cognition
Item 26	Concentration difficulty	ACDM	0.52	37.11	0.894	0.99	0.803	2.43	0.488	Cognition
Item 27	Pessimism about future	ACDM	0.91	41.29	0.743	5.90	0.207	1.33	0.856	Cognition
Item 28	Clear and quick thinking	GDINA	0.73	46.00	0.635	2.10	0.349	0.30	0.860	Cognition
Item 29	Judicious	GDINA	0.46	46.25	0.625	0.17	0.917	2.00	0.367	Cognition
Item 30	Desperation	ACDM	0.88	32.76	0.964	3.42	0.331	1.55	0.671	Cognition
Item 31	Disappointing	ACDM	0.76	66.10	0.052	2.01	0.570	3.10	0.376	Cognition
Item 32	Efforts are useless	RRUM	0.75	55.75	0.236	4.04	0.257	7.01	0.072	Cognition
Item 33	Past sorrow	ACDM	0.55	45.13	0.631	0.88	0.831	4.93	0.177	Cognition
Item 34	Sex with joy	ACDM	0.44	72.58	0.016	2.27	0.518	0.51	0.917	Mood
Item 35	Abandonment	ACDM	0.74	48.52	0.452	3.32	0.506	0.36	0.986	Mood
Item 36	Still depressed with others’ help	ACDM	0.75	54.02	0.288	0.59	0.898	1.69	0.640	Mood
Item 37	Inner mental collapsion	GDINA	0.81	43.58	0.654	3.34	0.912	2.97	0.936	Mood
Item 38	Satisfaction feelings	ACDM	0.74	49.40	0.457	3.34	0.342	0.37	0.946	Mood
Item 39	Fond of communication	RRUM	0.59	52.29	0.348	1.98	0.577	1.53	0.675	Mood
Item 40	Loss of interest	ACDM	0.72	69.46	0.029	0.37	0.946	2.56	0.464	Mood
Item 41	Early awakening	GDINA	0.52	43.90	0.715	0.77	0.680	4.14	0.126	Somatic
Item 42	Loss of appetite	GDINA	0.48	62.27	0.114	0.30	0.862	0.58	0.748	Somatic
Item 43	Awakening at nights	GDINA	0.51	57.70	0.212	0.02	0.990	1.40	0.496	Somatic
Item 44	Poor quality of sleep	GDINA	0.66	54.13	0.320	0.65	0.721	1.77	0.412	Somatic
Item 45	Poor sleep or somnolence	GDINA	0.59	49.58	0.490	1.43	0.489	2.07	0.356	Somatic
Item 46	Dizziness	GDINA	0.62	58.33	0.196	1.30	0.522	0.21	0.900	Somatic
Item 47	Good appetite	GDINA	0.93	74.64	0.014	1.81	0.404	0.92	0.632	Somatic
Item 48	Unchanged appetite	RRUM	0.82	71.69	0.019	3.72	0.293	0.72	0.869	Somatic
Item 49	Insomnia-early	GDINA	0.54	57.35	0.221	1.58	0.453	0.22	0.897	Somatic
Item 50	Suicidal thoughts	ACDM	0.83	41.60	0.765	3.94	0.268	0.90	0.827	Suicidality
Item 51	Inability to continue	ACDM	0.86	50.01	0.433	0.53	0.911	1.02	0.796	Suicidality
Item 52	Hardship feelings	ACDM	0.77	42.26	0.741	6.69	0.083	2.46	0.483	Suicidality
Item 53	Planning suicide	GDINA	0.64	37.90	0.895	0.42	0.811	1.50	0.473	Suicidality
Item 54	Wish was dead	GDINA	0.81	36.23	0.928	0.11	0.947	0.94	0.626	Suicidality
Item 55	Life is meaningful	ACDM	0.88	60.72	0.086	2.31	0.805	5.38	0.371	Suicidality
Item 56	Others’ life will be better without me	ACDM	0.69	39.02	0.845	1.09	0.780	2.42	0.490	Suicidality

*Disc., discrimination; DIF1, DIF (Male vs. Female); DIF2, DIF(Rural vs. Urban); RRUM, the Reduced Reparameterized Unified Model; G-DINA, the general DINA model; A-CDM, the additive CDM.*

### Reliability and Validity

Classification consistency refers to the extent to which participant classifications agree between two independent administrations, which is also called the reliability of classifications (Cui et al., 2012). As shown in Table 5, all attributes have classification consistency greater than 0.95 which suggests the CDMs-D has high reliability of classifications. In addition, classification accuracy refers to the extent to which the participants’ classifications agree with their true latent classes (Cui et al., 2012). Table 5 showed that the CDMs-D had high probability of classifying participants accurately based on their observed responses since all attributes have classification accuracy greater than 0.94.

**Table 5:** The reliability and validity of the CDMs-D.

CDMs-D	Classification consistency	Classification accuracy	Test score of CDMs-D	SDS	Self-reported depression
Test score of CDMs-D	—	—	1	0.810^∗∗∗^	0.707^∗∗∗^
Screening assessments	—	—	0.902^∗∗∗^	0.791^∗∗∗^	0.651^∗∗∗^
C1	0.983	0.973	0.827^∗∗∗^	0.689^∗∗∗^	0.621^∗∗∗^
C2	0.961	0.978	0.669^∗∗∗^	0.692^∗∗∗^	0.516^∗∗∗^
C3	0.973	0.960	0.805^∗∗∗^	0.699^∗∗∗^	0.590^∗∗∗^
C4	0.958	0.938	0.705^∗∗∗^	0.722^∗∗∗^	0.470^∗∗∗^
C5	0.962	0.958	0.763^∗∗∗^	0.603^∗∗∗^	0.486^∗∗∗^
C6	0.970	0.955	0.776^∗∗∗^	0.647^∗∗∗^	0.503^∗∗∗^
C7	0.962	0.959	0.765^∗∗∗^	0.742^∗∗∗^	0.552^∗∗∗^
C8	0.987	0.973	0.705^∗∗∗^	0.621^∗∗∗^	0.539^∗∗∗^
C9	0.965	0.945	0.624^∗∗∗^	0.563^∗∗∗^	0.464^∗∗∗^
C10	0.977	0.952	0.583^∗∗∗^	0.608^∗∗∗^	0.445^∗∗∗^

*SDS, Zung’s Self-Rating Depression Scale (1965); CDS, Carroll’s Depression Scale (Carroll et al., 1981); CES-D, Center for Epidemiologic Studies Depression Scale (Radloff, 1977); C1–C10 represent 10 symptom criteria for depression defined in ICD-10 shown in Table 1; ^∗∗∗^represents p < 0.001. screening assessments were the probability of depressive disorder based on CDMs-D via CDM.*

**FIGURE 1:**
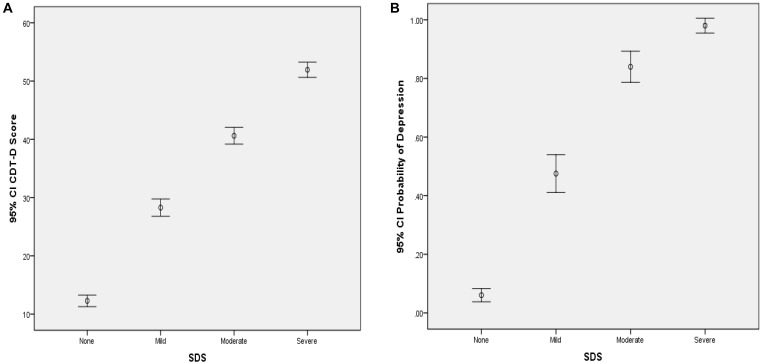
Error bar graph of the CDMs-D scores **(A)** and the probability of depressive disorder **(B)** for different groups via SDS. 95% CI, 95% confidence interval. The probability of depressive disorder (i.e., probability of mild, moderate and severe depression) was calculated based on the CDMs-D and the diagnostic criteria in *ICD-10* via CDMs.

From Table 4, the CDMs-D measures all depression symptoms defined in *ICD-10* and cover all five domains of depression defined by Gibbons et al. (2012), which implies that it has appropriate content validity. As for the criterion-related and convergent validity, the CDMs-D has a correlation of 0.707 (*p* < 0.001) and 0.810 (*p* < 0.001) with self-reported depression and SDS, respectively. The estimated probability of having mild, moderate or severe depression has a correlation of 0.791 (*p* < 0.001) and 0.651 (*p* < 0.001) with SDS and self-reported depression, respectively. Moreover, we calculated the coefficient of classification consistency between the CDMs-D and the structured clinical interview by psychotherapists via *ICD-10*, and results showed that there had a moderate coefficient of classification consistency with 0.463 (*p* < 0.001) between them. Figure 1, 2 show the 95% confidence intervals (CIs) for the mean CDMs-D score and the mean probability of having depressive disorder, respectively, for individuals with or without depression defined by the SDS or self-reported depression. Different groups have quite different mean CDMs-D scores and mean probabilities of depressive disorder, suggesting that the CDMs-D has the power to discriminate individuals with depression at different levels of severity.

**FIGURE 2:**
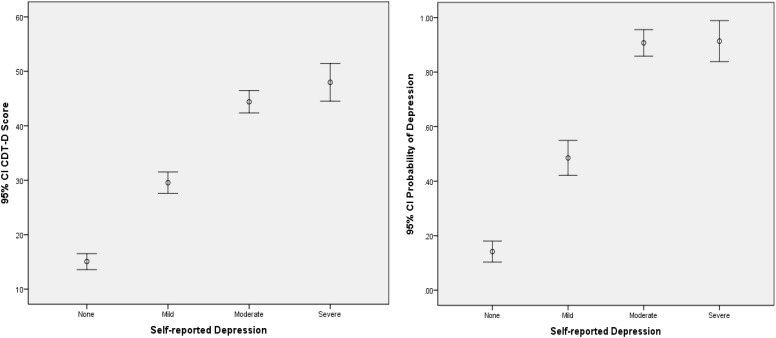
Error bar graph of the CDMs-D scores and the probability of depressive disorder for different groups via self-reported depression. 95% CI, 95% confidence interval. The probability of depressive disorder (i.e., probability of mild, moderate, and severe depression) was calculated based on the CDMs-D and the diagnostic criteria in *ICD-10* via CDMs.

### Screening Scores Reporting

**FIGURE 3:**
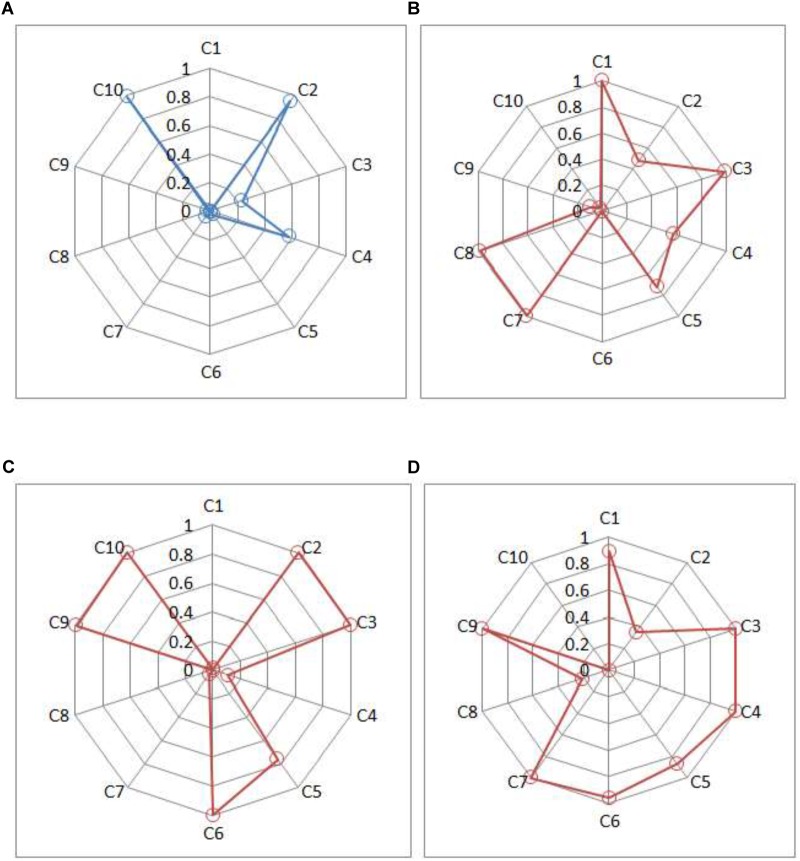
Symptom spectrum of depression for three patients and one healthy individual. **(A)** Individual A, **(B)** Patient B, **(C)** Patient C, and **(D)** Patient D. Criteria C1, C2, and C3 represent three typical symptoms; criteria C4–C10 represent seven common criteria in *ICD-10* in Table 1.

Compared with existing instruments for depression, CDMs-D could provide unique screening information for each patient. For illustration, score reports for four individuals (three patients and one healthy individual) were displayed in Figure 3. Three patients were chosen in that: (1) they were classified as moderate depression by their psychotherapists; (2) they had the same SDS score and were defined as moderate depression via the criterion of SDS; (3) they reported that they usually had considerable difficulty in continuing with social, work or domestic activities. Figure 3 shows the posterior probability that each criterion has been satisfied for these individuals. Based on these probabilities, the chances of having mild, moderate or severe depression for each individual can be calculated.

Individual A (male, 25 years old and from rural) has very high posterior probabilities of satisfying the typical symptom C2 and the common symptoms C10. Based on *ICD-10*, the estimated probabilities of being normal, mild, moderate and severe depression are 0.81, 0.12, 0.06, and 0.01, respectively, which suggests that it is unlikely for him to have depressive disorder.

Patients B, C, and D are all classified as having moderate depressive disorder by the CDMs-D (with the estimated posterior probability of 0.99, 0.99, 0.63, respectively), which is consistent to the results of their psychotherapists and SDS. However, they differ in their symptom profiles. From Figure 3, Patient B (female, 23 years old and from rural) probably satisfies two typical symptoms (C1 and C3) and four common symptoms (C4, C5, C7, and C8); Patient C (male, 29 years old and from rural) probably satisfies two typical symptoms (C1 and C3) and four common symptoms (C5, C6, C9, and C10); and Patient D (male, 58 years old and from urban) probably satisfies two typical symptoms (C1 and C3) and five common symptoms (C4, C5, C6, C7, and C9). Additionally, it can be seen that Patient B has a very high posterior probability of having symptom C8 (ideas or acts of self-harm or suicide) but Patient C and Patient D have very low probabilities. The information of symptom spectrum of each individual as showed in Figure 3 give insight into tailoring individual-specific treatments for depression. For example, for Patient B, the targeted treatment should focus on decreasing the chance of having ideas or acts of self-harm or suicide, for Patient C the targeted treatment should aim to decrease the fatigability and improve the enjoyment, while for Patient D, helping her to establish a brief of bring future is very important for him.

## Discussion and Conclusion

In this article, a new instrument for depression, the CDMs-D, is developed under the CDM framework based on *ICD-10*. This is the first study to measure the depressive disorder from the CDM perspective, though CDMs have been used as psychometric tools to analyze patient-reported outcomes, such as the pathological gambler in Templin and Henson (2006), neurocognitive functions in schizophrenia in Jaeger et al. (2006), internet addition in Tu et al. (2017) and the Millon Clinical Multiaxial Inventory-III in de la Torre et al. (2017). CDMs provide a set of psychometric tools to assess item properties, test reliability (Cui et al., 2012) and validity, and in this study, the CDMs-D with 56 items has been shown to have good reliability and validity. Comparing with the existing self-report measures (such as SDS, CES-D), one outstanding advantage of the new measure is that it measures all symptom criteria defined in the *ICD-10* and can provide symptom level reports. In addition, the high correlation between the CDMs-D and SDS indicated that the general-level information of depression they provided were high consistent. However the CDMs-D can provide the additional symptom-level information of depression. This dues to that the CDMs have the unique feature that can provide rich information in terms of whether the participants have met each symptom and of estimating the probability of having mild, moderate, and severe depressive disorder. Such information tends to be superior to the decision made based on total scores from some existing questionnaire in that it is obtained according to the ICD-10.

The proposed measure also has some latent contributions for the specifically assessing/screening for *ICD* and *DSM*-based depression. For example, this proposed measure aims to screen and monitor ICD and DSM-based depression, therefore it may provide a beneficial supplement to a clinician, especially when the patients cannot clearly and directly report whether all the symptoms defined in DSM or ICD are present. Another latent contribution is that it may reduce the burden of a clinician when there are large subjects for screening or monitoring. Moreover, a patient can conveniently make a self-examination about *ICD* and *DSM*-based depression by using the CDMs-D. Finally, a clinician can use the information from the measure, the clinical interview and others together to make diagnosis.

It is the CDMs that make these inferences possible, but the CDMs need to be used with cautions. Unlike classical test theory, factor analysis and IRT models, CDMs typically assume that latent variables are binary (Rupp et al., 2010). Because of this assumption, CDMs lend themselves well to modeling symptoms for many disorders in psychiatry. However, it is reasonable to ask whether the symptoms are binary or not in nature. It should be noted that all psychometric models, including CDMs, are just approximations of the real world, and therefore, as long as the symptoms can be approximately treated as binary variables especially for the ICD and DSM-based assessment of depression, the inferences can be useful. Additionally, CDMs consider the complex interactions among latent binary variables (de la Torre, 2011; Templin and Bradshaw, 2014) (e.g., unobserved symptoms). This, on one hand, allows greater flexibility than most IRT models in modeling item responses; but, on the other hand, tends to make the model complex with, sometimes, too many parameters. This study considered simplifying the saturated CDM with all possible interactions to some reduced models with fewer parameters to obtain more stable parameter estimates. These analyses are important because, in general, a simpler model should be preferred to a complicated model if both fit data well.

Despite promising results, to unlock the potential of the CDMs, more research is needed. First, the current CDMs-D with 56 items is relatively long. It is important to consider a shorter version of CDMs-D to decrease patients’ test burden (Smiits et al., 2011). The computerized adaptive testing (CAT) may be an option to decrease the test length without a loss of measurement precision. Some research on combining CDM and CAT can be found in literature in the field of psychometrics (e.g., Cheng, 2009), but applications are lagging behind. Therefore, further research may empirical investigate how to amalgamate CDMs and CAT (CD-CAT; Cheng, 2009; Wang et al., 2011) to develop the CAT version of CDMs-D. Second, the outputs with probabilities of the proposed measure may be not familiar and accustomed for users. For example, this CDT-T may provide two types of probabilities: one is the probabilities of none depression, mild depression, moderate depression and severe depression, which add up to 100%; another is the probability of presence for each symptom. The former probabilities can be used as screening or monitoring while the latter probabilities can be used to investigate the symptoms characteristic for each patient. That is to say this measure can provide both general level and symptom level information. Third, this article considered the symptom criteria for depression defined in *ICD-10*, future research may explore whether it is appropriate to use the criteria defined in *DSM-5*. Fourth, future study should compare the CDMs-D and the structured interview protocols based on either the *ICD-10* or the *DSM-5*. Fifth, except of results in CDMs-D, other evidences such as a structured clinical interview should also be taken full consideration to give a diagnosis of depression. Sixth, there are also some commonly used dimensional measures of depression that are not included in this article, therefore more measures should be considered for future study. Last, the selected CDMs in this study involve a large number of parameters. The sample used for test calibration may not be large enough and therefore, some statistical procedures such as the Wald test for model selection and DIF detection may be affected due to poorly estimated covariance matrix (Philipp et al., 2017). Larger sample should be considered to stabilize the parameter estimation.

## Data Availability

The raw data supporting the conclusions of this manuscript will be made available by the authors, without undue reservation, to any qualified researcher.

## Ethics Statement

This study was carried out in accordance with the recommendations of ethics committee of Center for Mental Health Education and Research of Jiangxi Normal University with written informed consent from all subjects. All subjects gave written informed consent in accordance with the Declaration of Helsinki. The protocol was approved by the ethics committee of Center for Mental Health Education and Research of Jiangxi Normal University.

## Author Contributions

DW contributed to thesis writing and code writing. XG processed the data. YC performed to guide the data processing and code writing. DT contributed to guide the thesis writing and code writing.

## Conflict of Interest Statement

The authors declare that the research was conducted in the absence of any commercial or financial relationships that could be construed as a potential conflict of interest.
